# Supplementary material for: Differential Effects of Lipid Bilayers on αPSM Peptide Functional Amyloid Formation
Source: Int J Mol Sci. 2023 Dec 20;25(1):102. doi: 10.3390/ijms25010102 (PMC10779341; doi:10.3390/ijms25010102)
Supplement: Supplementary file 1 [file ijms-25-00102-s001.zip › ijms-2727397-supplementary.pdf]

## Supplementary

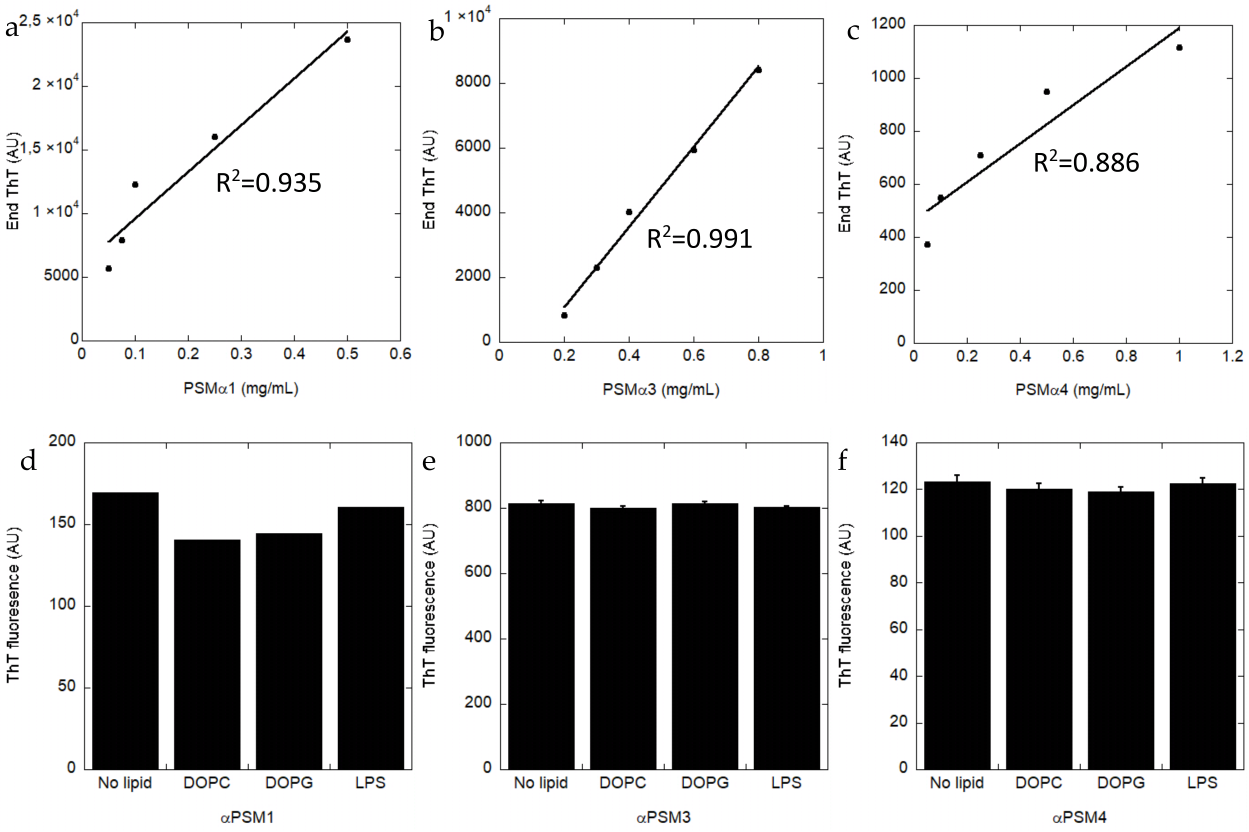


**Figure S1**. Experimental controls. (**a**) End ThT fluorescence signal for αPSM1. (**b**) End ThT fluorescence signal for αPSM3. (**c**) End ThT fluorescence signal for αPSM4. (**d**) ThT fluorescence signal of preformed αPSM1 aggregates (0.25 mg/mL) in the presence and absence of 100 µg/mL DOPC, DOPG, and LPS lipid vesicles. (**e**) ThT fluorescence signal of preformed αPSM3 aggregates (0.25 mg/mL) in the presence and absence of 100 µg/mL DOPC, DOPG, and LPS lipid vesicles. (**f**) ThT fluorescence signal of preformed αPSM4 aggregates (0.25 mg/mL) in the presence and absence of 100 µg/mL DOPC, DOPG, and LPS lipid vesicles.

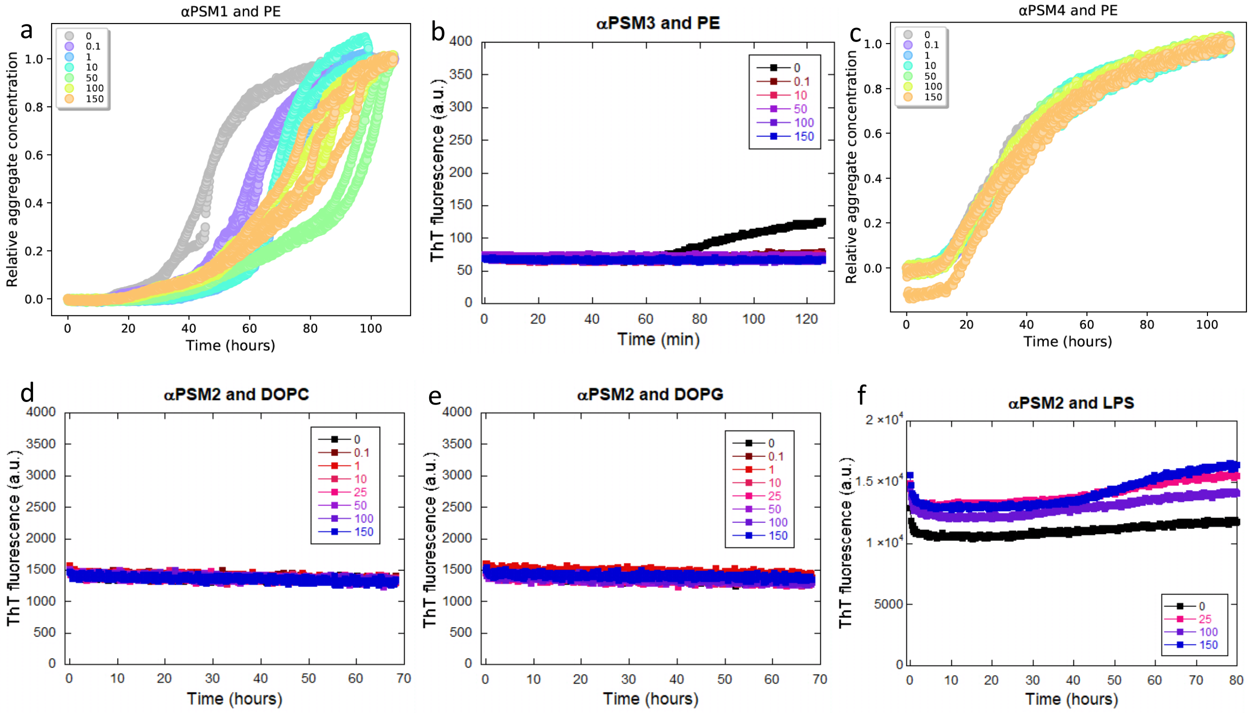


**Figure S2.** Additional experimental kinetic results. (**a**) Normalized kinetic data with $\alpha$PSM1 (0.25 mg/mL) added PE (concentration stated in µg/mL). (**b**) Raw kinetic data with $\alpha$PSM3 (0.5 mg/mL) added PE (concentration stated in µg/mL). (**c**) Normalized kinetic data with $\alpha$PSM4 (0.25 mg/mL) added PE (concentration stated in µg/mL). All kinetic experiments were carried out in triplicates. Please note the differences in the y- and x-axis between the figures. (**d**) Raw kinetic data with $\alpha$PSM2 (0.25 mg/mL) added DOPC (concentration stated in µg/mL). (**e**) Raw kinetic data with $\alpha$PSM2 (0.25 mg/mL) added DOPG (concentration stated in µg/mL). (**f**) Raw kinetic data with $\alpha$PSM2 (0.25 mg/mL) added LPS (concentration stated in µg/mL).

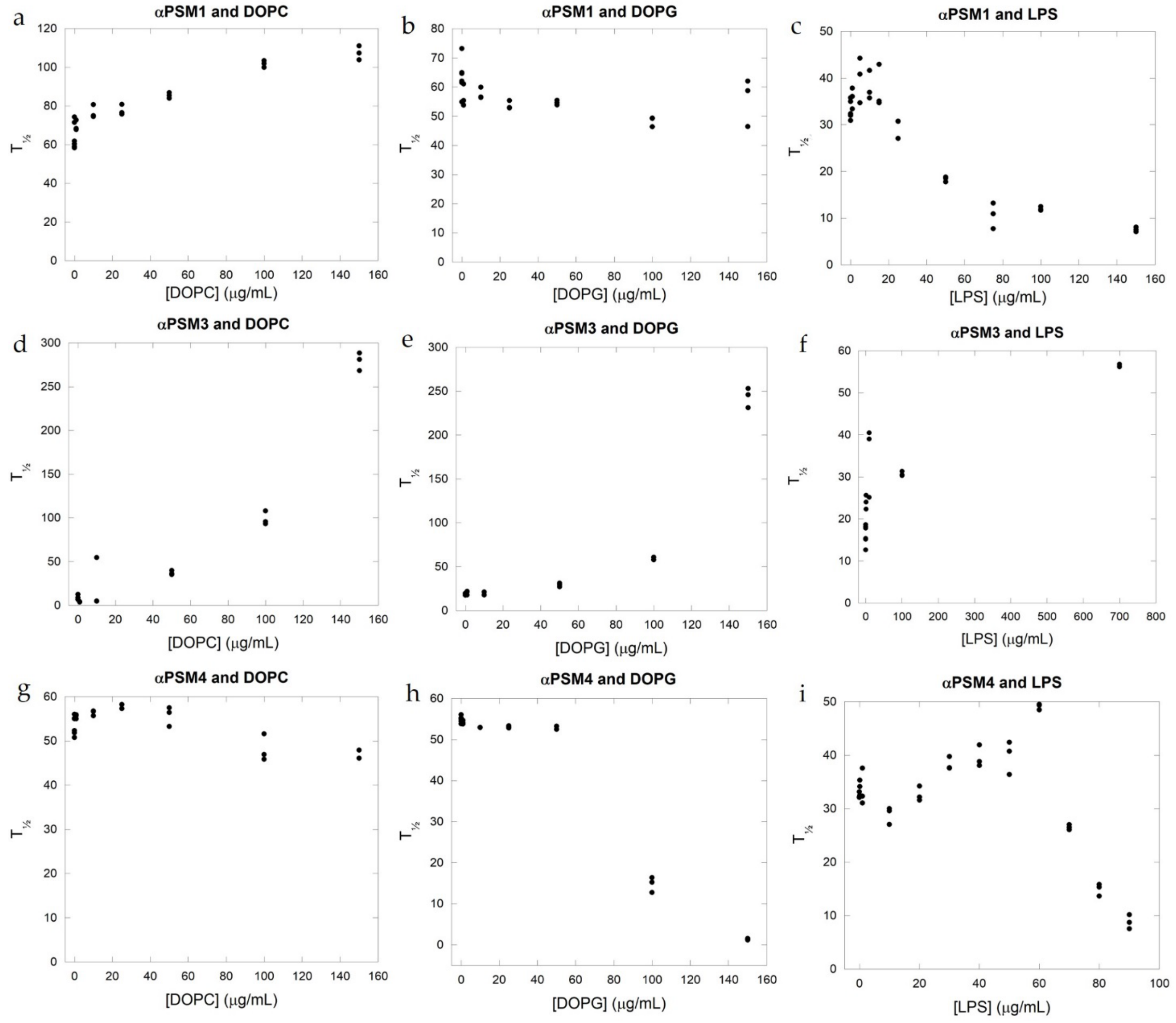


**Figure S3.** Half-time plots for $\alpha$PSM1, $\alpha$PSM3 and $\alpha$PSM4 when added various concentrations of lipids. The half-times are a function of lipid concentration in μg/mL obtained from the three repeats of the aggregation experiments for each peptide and lipid combinations. (**a**) $\alpha$PSM1 and DOPC (**b**) $\alpha$PSM1 and DOPG (**c**) $\alpha$PSM1 and LPS (**d**) $\alpha$PSM3 and DOPC (**e**) $\alpha$PSM3 and DOPG (**f**) $\alpha$PSM3 and LPS (**g**) $\alpha$PSM4 and DOPC (**h**) $\alpha$PSM4 and DOPG (**i**) $\alpha$PSM4 and LPS. Please note the differences in the y-axis.

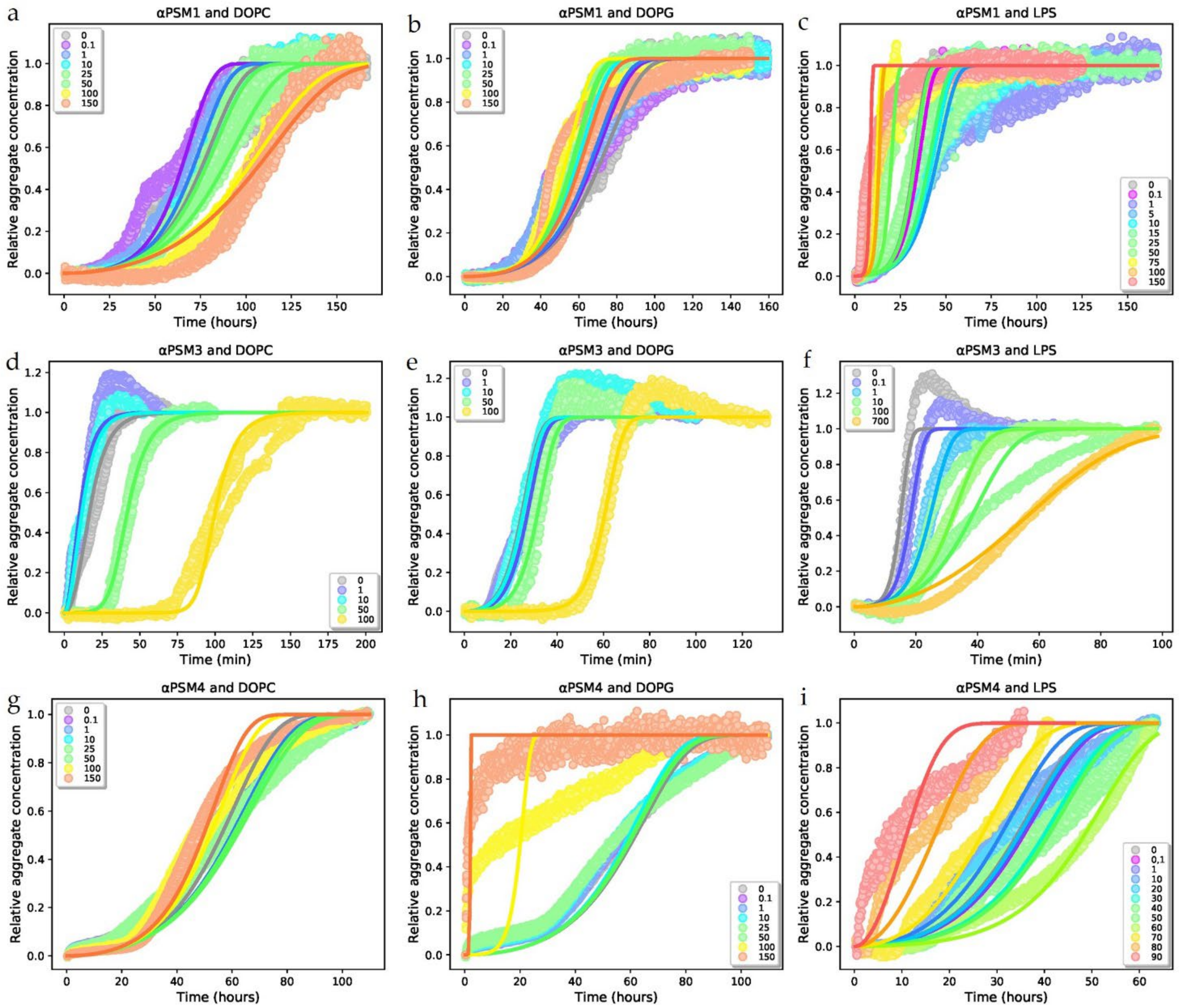


**Figure S4.** Fitting of aggregation kinetic data for PSM peptides in the presence of lipids using Amylofit. All kinetic data is fitted to a secondary nucleation-dominated model. (**a**) $\alpha$PSM1 in the presence of DOPC lipid vesicles fitted to a secondary nucleation model ($k_+k_2$ as fitting parameter and $k_+k_n$ as a global fit). (**b**) $\alpha$PSM1 in the presence of DOPG lipid vesicles fitted to a secondary nucleation model ($k_+k_2$ as fitting parameter and $k_+k_n$ as a global fit). (**c**) $\alpha$PSM1 in the presence of LPS lipid vesicles fitted to a secondary nucleation model ($k_+k_2$ as fitting parameter). (**d**) $\alpha$PSM3 in the presence of DOPC lipid vesicles fitted to a secondary nucleation model ($k_+k_n$ as fitting parameter). (**e**) $\alpha$PSM3 in the presence of DOPG lipid vesicles fitted to a secondary nucleation model ($k_+k_n$ as fitting parameter and $k_+k_2$ as a global fit). (**f**) $\alpha$PSM3 in the presence of LPS lipid vesicles fitted to a secondary nucleation model ($k_+k_2$ as fitting parameter). (**g**) $\alpha$PSM4 in the presence of DOPC lipid vesicles fitted to a secondary nucleation model ($k_+k_2$ as fitting parameter). (**h**) $\alpha$PSM4 in the presence of DOPG lipid vesicles fitted to a secondary nucleation model ($k_+k_2$ as fitting parameter). (**i**) $\alpha$PSM4 in the presence of LPS lipid vesicles fitted to a secondary nucleation model ($k_+k_n$ as fitting parameter). Parameters from the data fitting are shown in **Table S1.**

**Table S1:** Kinetic parameters from fitting of kinetic data of PSM peptide in the presence of DOPC, DOPG and LPS lipid vesicles.

| αPSM1 | | | | | |
|---|---|---|---|---|---|
| [DOPC] (µg/mL) | $k_+k_2$ ($M^{-n_c-1}\ h^{-2}$) | [DOPG] (µg/mL) | $k_+k_2$ ($M^{-n_c-1}\ h^{-2}$) | [LPS] (µg/mL) | $k_+k_2$ ($M^{-n_c-1}\ h^{-2}$) |
| 0 | 15.4 | 0 | 14.9 | 0 | 154 |
| 0.1 | 26.5 | 0.1 | 18.4 | 0.1 | 123 |
| 1 | 19.3 | 1 | 19.7 | 1 | 67.8 |
| 10 | 13.9 | 10 | 29.3 | 5 | 55.8 |
| 25 | 13.8 | 25 | 34.6 | 10 | 68.3 |
| 50 | 8.78 | 50 | 33.2 | 15 | 75.8 |
| 100 | 4.72 | 100 | 42.0 | 25 | 148 |
| 150 | 3.74 | 150 | 26.4 | 50 | 617 |
| | | | | 75 | $2.31 \times 10^{3}$ |
| | | | | 100 | $1.84 \times 10^{3}$ |
| | | | | 150 | $5.91 \times 10^{3}$ |
| $m_o$ | 111 µM | $m_o$ | 111 µM | $m_o$ | 111 µM |
| $k_+k_n$ | $3.03 \times 10^{-5}$ ($M^{-n_c}\ h^{-2}$) global fit | $k_+k_n$ | $4.32 \times 10^{-5}$ ($M^{-n_c}\ h^{-2}$) global fit | $k_+k_n$ | $6.98 \times 10^{-5}$ ($M^{-n_c}\ h^{-2}$) |
| $n_c$ | $7.84 \times 10^{-6}$ | $n_c$ | $7.84 \times 10^{-6}$ | $n_c$ | $7.84 \times 10^{-6}$ |
| $n_2$ | $1.66 \times 10^{-3}$ | $n_2$ | $1.66 \times 10^{-3}$ | $n_2$ | $1.66 \times 10^{-3}$ |
| MRE | $5.23 \times 10^{-3}$ | MRE | $6.05 \times 10^{-3}$ | MRE | $1.19 \times 10^{-2}$ |
| **αPSM3** | | | | | |
| [DOPC] (µg/mL) | $k_+k_n$ ($M^{-n_c}\ min^{-2}$) | [DOPG] (µg/mL) | $k_+k_n$ ($M^{-n_c}\ min^{-2}$) | [LPS] (µg/mL) | $k_+k_2$ ($M^{-n_c-1}\ min^{-2}$) |
| 0 | $1.86 \times 10^{12}$ | 0 | 1.44 | 0 | $1.52 \times 10^{3}$ |
| 1 | $9.16 \times 10^{12}$ | 1 | 0.915 | 0.1 | 868 |
| 10 | $6.13 \times 10^{12}$ | 10 | 1.29 | 1 | 352 |
| 50 | $6.51 \times 10^{9}$ | 50 | 0.401 | 10 | 75.8 |
| 100 | $1.64 \times 10^{4}$ | 100 | $1.87 \times 10^{-3}$ | 100 | 147 |
| | | | | 700 | 11.2 |
| $m_o$ | 192 µM | $m_o$ | 192 µM | $m_o$ | 192 µM |
| $k_+k_n$ | - | $k_+k_n$ | - | $k_+k_n$ | $3.01 \times 10^{-1}$ ($M^{-n_c}\ min^{-2}$) global fit |
| $k_+k_2$ | $1.80 \times 10^{13}$ ($M^{-n_c-1}\ min^{-2}$) | $k_+k_2$ | 255 ($M^{-n_c-1}\ min^{-2}$) global fit | $k_+k_2$ | - |
| $n_c$ | 4.00 | $n_c$ | 1.00 | $n_c$ | 0.600 |
| $n_2$ | 3.00 | $n_2$ | 0.123 | $n_2$ | 0.123 |
| MRE | $6.38 \times 10^{-3}$ | MRE | $5.36 \times 10^{-3}$ | MRE | $7.55 \times 10^{-3}$ |
| **αPSM4** | | | | | |
| [DOPC] (µg/mL) | $k_+k_2$ ($M^{-n_c-1}\ h^{-2}$) | [DOPG] (µg/mL) | $k_+k_2$ ($M^{-n_c-1}\ h^{-2}$) | [LPS] (µg/mL) | $k_+k_n$ ($M^{-n_c}\ h^{-2}$) |
| 0 | 49.3 | 0 | 47.6 | 0 | $2.50 \times 10^{-4}$ |
| 0.1 | 37.9 | 0.1 | 50.4 | 0.1 | $2.16 \times 10^{-4}$ |
| 1 | 38.0 | 1 | 52.2 | 1 | $2.22 \times 10^{-4}$ |
| 10 | 35.7 | 10 | 55.3 | 10 | $3.60 \times 10^{-4}$ |
| 25 | 32.5 | 25 | 51.6 | 20 | $2.39 \times 10^{-4}$ |
| 50 | 36.0 | 50 | 50.4 | 30 | $1.40 \times 10^{-4}$ |
| 100 | 66.3 | 100 | $1.61 \times 10^{3}$ | 40 | $1.27 \times 10^{-4}$ |
| 150 | 79.5 | 150 | $5.32 \times 10^{5}$ | 50 | $1.26 \times 10^{-4}$ |
| | | | | 60 | $5.92 \times 10^{-5}$ |

| | | | | | |
|---|---|---|---|---|---|
| | | | | 70 | 5.58 x $10^{-4}$ |
| | | | | 80 | 1.92 x $10^{-3}$ |
| | | | | 90 | 5.26 x $10^{-3}$ |
| $m_o$ | 45.4 µM | $m_o$ | 40.2 µM | $m_o$ | 45.4 µM |
| $k_+k_n$ | 7.79 x $10^{-5}$ $(M^{-n_c}\ h^{-2})$ | $k_+k_n$ | 7.00 x $10^{-5}$ $(M^{-n_c}\ h^{-2})$ | $k_+k_n$ | - |
| $k_+k_2$ | - | $k_+k_2$ | - | $k_+k_2$ | 130 $(M^{-n_c-1}\ h^{-2})$ |
| $n_c$ | 8.00 x $10^{-6}$ | $n_c$ | 8.00 x $10^{-6}$ | $n_c$ | 8.00 x $10^{-5}$ |
| $n_2$ | 1.70 x $10^{-3}$ | $n_2$ | 1.70 x $10^{-3}$ | $n_2$ | 2.00 x $10^{-2}$ |
| MRE | 4.45 x $10^{-3}$ | MRE | 1.38 x $10^{-2}$ | MRE | 7.17 x $10^{-3}$ |

**Table S2.** Wavelength minimum for CD spectra of $\alpha$PSM peptide fibril samples in the absence and presence of lipid vesicles.

| Peptide | DOPC | $\lambda_{min}$ (nm) | DOPG | $\lambda_{min}$ (nm) | LPS | $\lambda_{min}$ (nm) |
|---|---|---|---|---|---|---|
| **αPSM1** | 0 µg/mL | 219 | 0 µg/mL | 219 | 0 µg/mL | 219 |
| | 50 µg/mL | 218.5 | 10 µg/mL | 217.5 | 50 µg/mL | 217.5 |
| | 150 µg/mL | 218.5 | 150 µg/mL | 218.5 | 150 µg/mL | 218 |
| **αPSM3** | 0 µg/mL | 224 | 0 µg/mL | 224 | 0 µg/mL | 224 |
| | 50 µg/mL | 226 | 10 µg/mL | 226.4 | 10 µg/mL | 225.5 |
| | 150 µg/mL | 227.8 | 150 µg/mL | 224.6 | 700 µg/mL | 213.5 |
| **αPSM4** | 0 µg/mL | 216 | 0 µg/mL | 216 | 0 µg/mL | 216 |
| | 25 µg/mL | 127.9 | 10 µg/mL | 126.8 | 10 µg/mL | 215.5 |
| | 150 µg/mL | 217.9 | 150 µg/mL | 226.9 | 90 µg/mL | 222.5 |

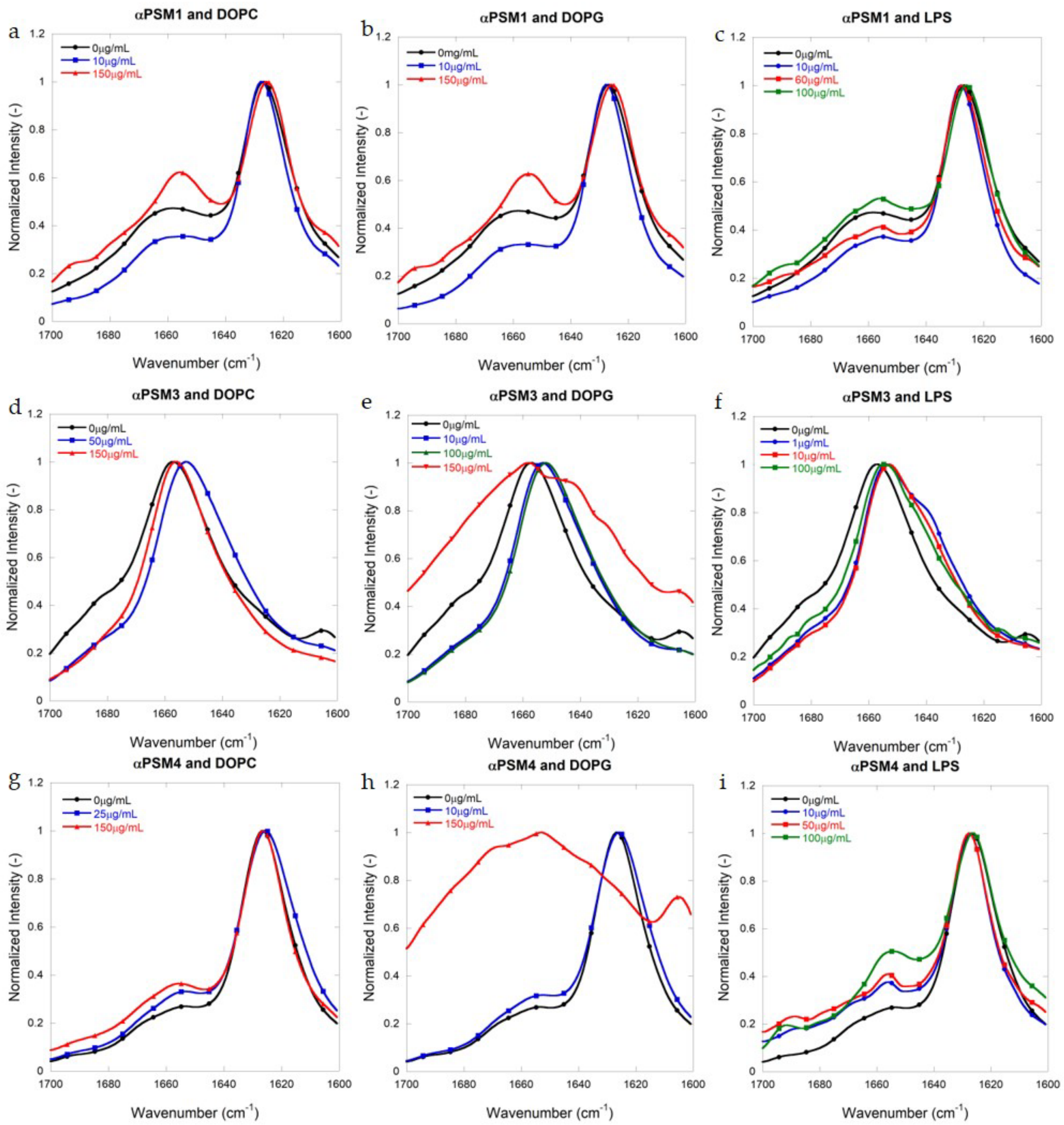


**Figure S5.** Fourier transform infrared (FTIR) spectroscopy of the amide I′ region (1600–1700 cm$^{-1}$) of fibrils of PSMs variants. (**a**) PSM$\alpha$1 and DOPC. (**b**) PSM$\alpha$1 and DOPG. (**c**) PSM$\alpha$1 and LPS. (**d**) PSM$\alpha$3 and DOPC. (**e**) PSM$\alpha$3 and DOPG. (**f**) PSM$\alpha$3 and LPS. (**g**) PSM$\alpha$4 and DOPC. (**h**) PSM$\alpha$4 and DOPG. (**i**) PSM$\alpha$4 and LPS.

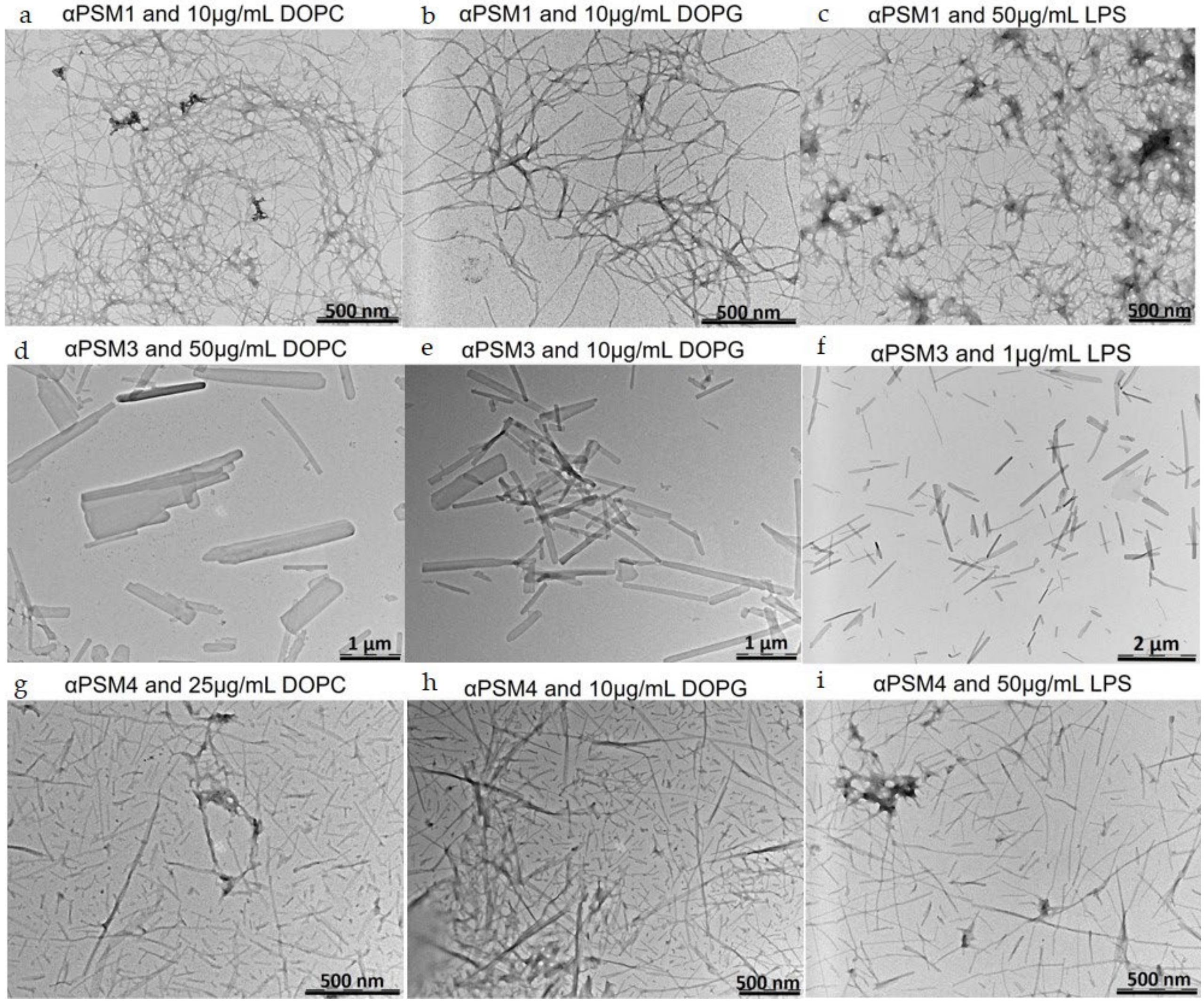


**Figure S6.** Morphology of aggregates of $\alpha$PSM peptides with low concentrations of lipids. Transmission electron microscopic images of the end state of PSM peptide fibril samples with DOPC, DOPG and LPS lipid vesicles. (**a**) $\alpha$PSM1 and 10 μg/mL DOPC. (**b**) $\alpha$PSM1 and 10 μg/mL DOPG. (**c**) $\alpha$PSM1 and 50 μg/mL LPS. (**d**) $\alpha$PSM3 and 50 μg/mL DOPC. (**e**) $\alpha$PSM3 and 10 μg/mL DOPG. (**f**) $\alpha$PSM1 and 1 μg/mL LPS. (**g**) $\alpha$PSM4 and 25 μg/mL DOPC. (**h**) $\alpha$PSM4 and 10 μg/mL DOPG. (**i**) $\alpha$PSM4 and 50 μg/mL.

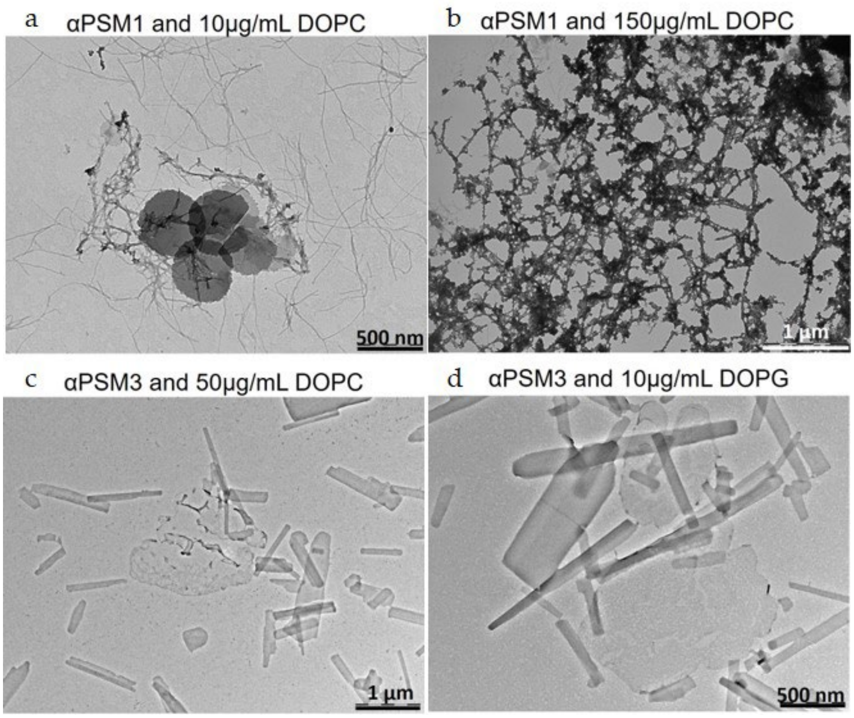


**Figure S7.** TEM images of lipid vesicles embedded in fibril formation. (**a**) $\alpha$PSM1 and 10 μg/mL DOPC. (**b**) $\alpha$PSM1 and 150 μg/mL DOPC. (**c**) $\alpha$PSM3 and 50 μg/mL DOPC. (**d**) $\alpha$PSM3 and 10 μg/mL DOPG.
